# Surgical publications: detecting and preventing fraud

**DOI:** 10.1111/ans.17579

**Published:** 2022-08-11

**Authors:** Michalis Koullouros, Guy Maddern

**Affiliations:** ^1^ Division of Surgery The Queen Elizabeth Hospital Adelaide South Australia Australia; ^2^ Surgical Specialties University of Adelaide, The Queen Elizabeth Hospital Adelaide South Australia Australia

Medical practitioners belong to a privileged profession which is committed to continuous professional development. Surgery is one of the most competitive disciplines, and Royal Colleges expect their fellows to engage in activities such as postgraduate degrees, research and teaching.^1^ This observation holds true for all grades of surgeons; trainees undertake these activities to gain training and fellowship spots, where consultants pursue educational activities to improve their practice and achieve desired employment. Therefore, research is important to career progression.^2^, ^3^

Unfortunately, these expectations lead to academic inflation with degrees and publications seen as means to furthering one's prospects. As a result, paper retraction is at an all‐time high due to fraudulent research.^4^ This may be due to better detection rather than a higher incidence, as journals do more than ever before to detect the phenomenon. Fraudulence includes fabrication, plagiarism, authorship, conflicts of interest, impact factor and misconduct, and duplication or redundancy of research.^3^ By reviewing original articles in reputable surgical journals, a study concluded that out of 660 articles they examined, 147 were suspicious for redundancy, with 77% of the suspected papers not citing the index publication.^5^ With regards to the most severe types of fraud, a meta‐analysis revealed that up to 4.9% of scientists admitted to fabricating data, 33.7% were involved in ‘questionable practises’, and up to 33.2% observed misconduct.^6^ The scientists observing misconduct most frequently were medical scientists.^6^ This observation is aligned with a study which showed that 55.7% of newly appointed consultants in a range of clinical specialties observed some form of research misconduct, 5% admitted past personal misconduct and 18% were either willing or unsure if they were willing to commit research misconduct in the future. Surgeons were the group most likely to encounter misconduct.^2^

An infamous example is Banerjee who, incredibly, achieved 49 publications in his first 18 months as a junior doctor working as a surgical resident in a tertiary teaching hospital in England.^7^ Banerjee's volume of data and outstanding results led to suspicions by the laboratory staff who refused to be added as authors on his research. Staff kept raising concerns as he was describing experiments utilizing equipment and techniques he was not trained to perform, and reported using consumables in amounts never purchased by the lab. The lab supervisor kept dismissing these issues, and even when Banerjee admitted wrongdoing, the internal investigators failed to reprimand him for his misdeeds.^7^ This indicates the power losing research grants holds over institutions. A more recent example is Macchiarini who claimed to have revolutionized synthetic trachea surgery.^8^, ^9^, ^10^, ^11^ Due to his charisma and status of the institution he worked for – the Karolinska – he continued performing these operations for nearly a decade despite the terrible outcomes and patient harm.^12^, ^13^ He was only exposed when he assured his journalist fiancee that the Pope would be presiding over their wedding. Her suspicions of dishonesty were revealed on the international stage, empowering others who had concerns about his medical practice to come forward. Even when criticized about the mortality of these procedures, Macchiarini vehemently defended his findings using evidence published by himself or his co‐authors.^14^, ^15^, ^16^, ^17^, ^18^ It is worth noting that only some of his publications have been retracted.

Journals have implemented strategies to prevent fraud. Firstly, authors are required to confirm that the work they are submitting is accurate and their own. Most journals require co‐authors to validate the authenticity of the data, in an attempt to ensure that if the primary author is being fraudulent, others may not wish to be complicit.^4^ Plagiarism is arguably the misconduct most prone to detection due to software utilized by a range of organisations.^19^ Peer review is thought to be assisting in detecting misconduct. However, as journal editors and publications admit, the purpose of peer review is to separate irrelevant or meaningless research from potentially useful science, and not fraud detection.^3^, ^4^, ^19^, ^20^ In reality, the journal expects that the scientists involved have had to prove the ethical standing of the data by passing ethics and funding proposals.^4^ For a reviewer to conclude that data is fabricated, they would need to examine the raw data and perform their own statistical analyses, as some reputable journals now do.^4^ This is clearly a resource intensive process, while indicating to fellow scientists that their data is scrutinised.^19^, ^20^ It is also important to remember that journals – other than rejecting a study – have no disciplinary power.

Experienced reviewers can suspect fraudulent data based on the numbers used.^4^, ^19^ Suspicious data stem from human psychology and preferencing rounding numeric data to 0 s and 5 s, or ‘padding’ data with numbers inconsistent with Newcomb's law which dictates that the 10 numerals do not occur in equal frequency, with low numbers having a higher incidence.^4^, ^19^ Exceptional results are always highly suspicious. For example, a Duke University oncologist reported outstanding but unreproducible results for patient‐tailored therapies.^4^ This lead to an investigation into the index publication's raw data which yielded several intentional errors.^4^ Similarly when a prominent South African oncologist's outstanding results in breast cancer treatments were interrogated, they were obviously fraudulent.^21^ Research during the COVID‐19 pandemic and the race to a breakthrough observed similar trends with fraudulent publications in some of the most prestigious journals. A retracted study published at the Lancet showed increased morbidity and mortality when hydroxychloroquine or quinine were used.^22^ When readers raised concerns on the validity of the data, the journal attempted to investigate but the authors refused to disclose the full data set. The same group of authors published another – later‐retracted – paper at the New England Journal of Medicine ‘confirming’ cardiovascular disease as a risk factor for increased mortality from COVID‐19.^23^ In the latter publication, the authors themselves requested a retraction citing issues with raw data disclosure to the entirety of the authors and the journal team.^24^

Fraud and misconduct in research publications is a product of its environment with some clinicians neglecting their duty to uphold ethics to further their careers. Our duty of care does not end when we leave the hospital, and should be reflected daily in every aspect of our professional and personal lives. Journals should feel empowered to request raw data, flag misconduct on a cross‐journal shared platform, and report culprits to their relevant regulatory boards for further investigation and reprimanding. As readers, we must be vigilant and raise concerns when reading papers with outstanding results, especially when these have not been validated. We must endeavour aiding reviewers in their work to help safeguard the honesty of published research and protect our patients from corrupt science.
